# Association Between Programmed Death-Ligand 1 Expression and the Vascular Endothelial Growth Factor Pathway in Angiosarcoma

**DOI:** 10.3389/fonc.2018.00071

**Published:** 2018-03-22

**Authors:** Sanjay P. Bagaria, Zoran Gatalica, Todd Maney, Daniel Serie, Mansi Parasramka, Steven Attia, Murli Krishna, Richard W. Joseph

**Affiliations:** ^1^Department of Surgery, Division of General Surgery, Mayo Clinic, Jacksonville, FL, United States; ^2^Caris Life Sciences, Phoenix, AZ, United States; ^3^Department of Health Sciences Research, Mayo Clinic, Jacksonville, FL, United States; ^4^Department of Cancer Biology, Mayo Clinic, Jacksonville, FL, United States; ^5^Department of Medicine, Division of Hematology/Oncology, Mayo Clinic, Jacksonville, FL, United States; ^6^Department of Pathology, Mayo Clinic, Jacksonville, FL, United States

**Keywords:** angiosarcoma, programmed death-ligand 1, immune microenvironment, checkpoint pathway, vascular endothelial growth factor

## Abstract

Angiosarcoma is a vascular malignancy associated with a poor prognosis and chemotherapy resistance. The tumor immune microenvironment of angiosarcoma has not been characterized. We investigated the expression of programmed death-ligand 1 (PD-L1) and programmed death 1 (PD-1) in angiosarcoma and correlated these findings with vascular endothelial growth factor (VEGF)-related gene expression and survival. Using archived formalin-fixed paraffin-embedded tissues of primary and metastatic angiosarcoma specimens, we characterized the immunohistochemical (IHC) expression of PD-L1 and PD-1. In addition, we extracted RNA from each tumor and quantified the expression of VEGF-related genes, and then tested if these genes were associated with PD-L1 and PD-1 expression and clinical outcomes. Retrospective review identified 27 angiosarcoma specimens collected between 1994 and 2012. IHC expression of tumor PD-L1, tumor-infiltrating immune cell PD-L1, and tumor-infiltrating immune cell PD-1 expression was identified in 5 (19%), 9 (33%), and 1 (4%) specimens, respectively. Expression of PD-L1 and PD-1 was not associated with VEGF-related gene expression or survival. PD-L1 tumor and tumor-infiltrating immune cells expression was identified in a large proportion of patients. Though neither was associated with VEGF-related gene expression or prognosis, targeting PD-1/PD-L1 may be of benefit for a significant proportion of angiosarcomas that do not respond to surgery, chemotherapy, or radiation.

## Introduction

Angiosarcoma is an aggressive vascular soft tissue sarcoma thought to arise from mesenchymal cells (1). Despite a multimodality approach, 5-year overall survival is 35% and there has been little progress in the management of this disease (2). The pathogenesis of angiosarcomas is likely related to defects in angiogenesis. Vascular endothelial growth factor (VEGF) is overexpressed in angiosarcomas when compared to benign vascular tissue, yet anti-VEGF drugs have failed to improve survival (1, 3). Therefore, there is a critical need to better understand the pathophysiology of angiosarcoma to help develop novel therapeutic strategies. One such strategy is to better understand angiosarcoma tumor microenvironment.

Extensive studies of the tumor microenvironment have demonstrated that tumors develop local mechanisms to evade immune system detection through the modulation of T cells (4). The presence of tumor-infiltrating lymphocytes (TILs) has been reported as a manifestation of this phenomenon and is associated with the prognosis of numerous cancers (5), and in particular, CD8+ TILs has been shown to have a positive effect on overall survival for patients with cutaneous angiosarcoma (6). Another key component of the tumor microenvironment and immune evasion is the expression of the immune checkpoint pathway programmed death 1 (PD-1) and programmed death-ligand 1 (PD-L1) (7). PD-L1 tumor expression can lead to T cell evasion and allow tumors to escape from anticancer immune system attack.

The tumor immune microenvironment of sarcomas is not well understood. Studies have suggested that sarcomas do express PD-L1 (8–11). These studies provide contrasting data on the incidence of PD-L1 angiosarcoma and its prognostic impact. Therefore, more studies on angiosarcomas are needed to better (1) characterize tumor PD-L1 expression and (2) determine if tumor PD-L1 expression plays a role in VEGF-related pathophysiology of this rare disease, and (3) study the association between tumor PD-L1 expression and prognosis.

Our group reported an inverse relationship between tumor PD-L1 expression and VEGF-related genes in renal cell carcinoma (12). We hypothesize a similar relationship that exists for angiosarcoma, a sarcoma subtype whose oncologic driver is likely related to the VEGF pathway. In this study, we measured the expression of PD-L1 and PD-1 in a large number of angiosarcoma specimens, correlate these findings with the expression of VEGF-related genes, and determine whether such expression is associated with overall survival.

## Materials and Methods

### Study Cohort

Retrospective review of the Mayo Clinic Jacksonville Cancer Database identified 44 unique patients with histological confirmation of angiosarcoma diagnosed between 1994 and 2010. All specimens were reviewed by a board-certified pathologist (MK) blinded to clinical results. Biopsy and surgical specimens were sufficient for analysis for a total 27 specimens from 25 patients. One patient had specimens from the primary and recurrence, and one patient had specimens from two separate sites of metastasis. We collected clinicopathologic data, including age, gender, histologic subtype, location, tumor type (primary, recurrence, metastasis), and receipt of radiation therapy. Mayo Clinic Institutional Review Board approved the study prior to collecting data and performing analysis. The study was deemed as minimal risk and, therefore, informed consent from study participants was not obtained.

### Characterization of PD-L1/PD-1 Expression

Formalin-fixed paraffin-embedded (FFPE) blocks were retrieved and 5 µm slides were reviewed by a board-certified pathologist (ZG) for immunohistochemical (IHC) expression of PD-L1 and PD-1 expression. The presence of PD-1+ tumor-infiltrating immune cells was evaluated using NAT105 monoclonal antibody (Ventana Medical Systems, Tucson, AZ, USA) and the average number of PD-1+ cells was assessed on 10 high (40× Olympus BX45 microscope) power fields (13). The presence of PD-L1+ tumor cells and tumor-infiltrating immune cells was evaluated using Caris Life Sciences (Phoenix, AZ, USA) validated laboratory developed IHC tests using SP-142 and SP-263 antibodies (Ventana). As previously described, tumor cells were considered PD-L1 positive if at least 5% of tumor cells expressed moderate membranous staining (≥2+), and tumor-infiltrating immune cells were considered PD-L1 positive if at least 5% of immune cells expressed weak membranous staining (≥1) (14).

Tumor infiltrating lymphocytes were examined on hematoxylin and eosin stained slides containing angiosarcoma. Slides were evaluated at ×400 magnification for the presence of TILs and if present, scored semi-quantitatively as either brisk or non-brisk using the protocol used by the College of American Pathologists (15).

### RNA Isolation From FFPE Samples and Nanostring Analysis

Total RNA was isolated from three slides per tumor at 10-µm sections of tumor-rich areas of FFPE tissue blocks using the AllPrep DNA/RNA FFPE kit reagents (Qiagen) following vendor’s standard protocols. Isolated FFPE RNA was treated with 20 units DNase I. RNA concentration was measured and purity analyzed on a NanoDrop ND-2000c Spectrophotometer (NanoDrop Technologies, Wilmington, DE, USA). RNA integrity was measured using the RNA 6000 Nano Assay on a 2100 Bioanalyzer (Agilent Technologies, Santa Clara, CA, USA). The NanoString platform was used to quantify gene expression of 27 candidate genes: ACAA2, ACAT1, BNIP3L, MAOB, NPR3, PD-1, PLEKHA7, SAA4, SLPI, ST13, TCEA3, TCN2, TPM4, B7H1, B7H3, BAP1, CTNNB1, CAIX, IMP3, KI-67, PBMR1, SETD2, SFPR1, TOP2A, VEGF, VEGFR1, and VEGFR2. 200 ng of each total RNA sample was prepared as per the manufacturer’s instructions. Gene expression was quantified on the NanoString nCounter™ and raw counts were generated with nSolver™. Raw counts per gene were extracted using the nSolver software (NanoString).

Count data were normalized as per NanoStriDE recommendations (16). Raw counts were corrected for positive and negative spike-in abundance, and then multiplied by a correction factor based on the sum of housekeeping genes. Resulting corrected counts were transformed to a log2 scale.

### Statistical Analysis

Linear mixed-effect models were employed to determine the association between PD-L1 and VEGF genes on the Nanostring panel, with patient serving as a random intercept. Cox models were generated to assess the relationship between gene expression in the primary tumor and overall survival from time of surgery, and the Kaplan–Meier method was used to visualize how outcomes differed between PD-L1 positive and negative patients. Univariate linear mixed-effect models were further used to investigate associations between patient characteristics and gene expression across the 27 genes assessed by Nanostring.

## Results

### Patient Characteristics

Of the 25 patients identified, the median age was 76.6 years (range 38–92) and 60% were male (Table 1). A total of 27 specimens were collected: 17 specimens were from primary tumors, 6 from recurrent tumors, and 4 from metastasis. Most of the tumors analyzed were high grade and cutaneous. Twelve patients were treated adjuvant radiation therapy. No patients were treated with neoadjuvant radiation therapy.

**Table 1:** Patient characteristics.

Characteristic	*N*	Percent
Total number of patients	25	
Total number of specimens	27	
Age, median, and years	76.6	
Male	15	60
Tumor type		
Primary	17	63
Recurrent	6	22
Metastasis	4	15
Histologic subtype		
Spindle-type	14	52
Epitheliod	13	48
Tumor size^a^, median, cm	2.0	
Grade		
Low	6	22
High	20	74
Missing	1	4
Cutaneous		
No	8	30
Yes	19	70
Primary location^a^		
Skin, scalp	4	24
Skin, trunk/extremities/face	10	59
Heart	2	7
Spleen	1	6

*^a^Primary tumors only*.

### PD-L1 and PD-1 Expression in Angiosarcoma

**Figure 1:**
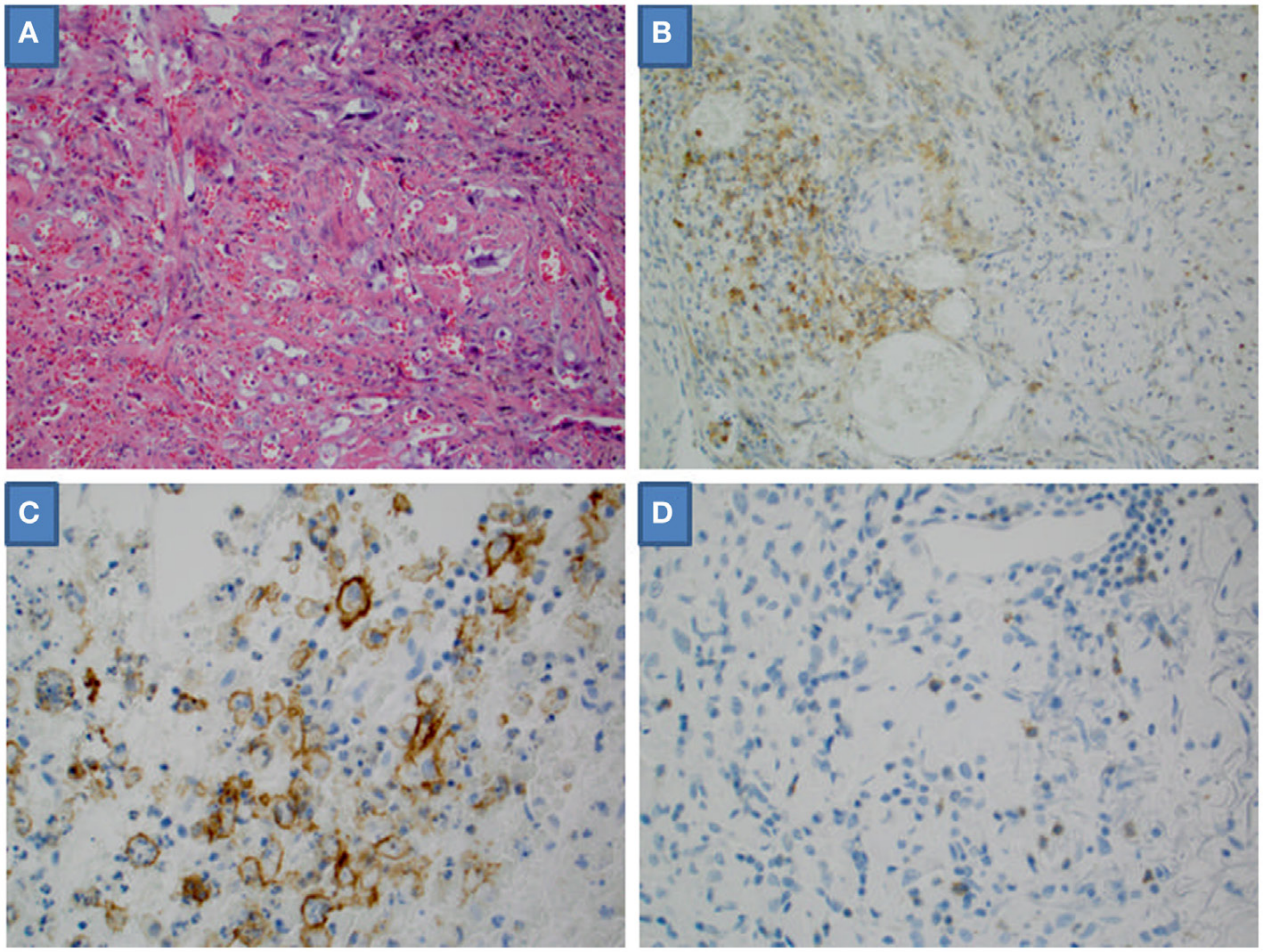
Microscopic high-powered view of angiosarcoma. **(A)** Hematoxylin and eosin stain. **(B)** Tumor-infiltrating immune cells positive for programmed death-ligand 1 (PD-L1). **(C)** Tumor cells positive for PD-L1. **(D)** Tumor-infiltrating immune cells positive for programmed death 1.

We examined the tumor microenvironment by quantifying PD-L1 and PD-1 IHC expression and measuring the presence of TILs (Figure 1). PD-L1 tumor expression was identified in 5 (19%) specimens, PD-L1 tumor-infiltrating immune cell expression in 9 (33%) specimens, and PD-1 tumor-infiltrating immune cell expression 1 (4%) specimens (Table 2). No differences were noted between the two antibodies used for PD-L1 IHC expression, neither in assessment of tumor cell nor tumor-infiltrating immune cell expression. TILs were absent in 2 (7%) of specimens. TILs were non-brisk and brisk in 19 (70%) and 6 (22%) specimens, respectively.

**Table 2:** Programmed death-ligand 1 (PD-L1) and programmed death 1 (PD-1) immunohistochemical staining.

Specimen	PD-1	PD-L1 (SP-142)				PD-L1 (SP-263)			
	Average/10 HPFs	Tumor (intensity, %)		Inflammatory/stromal cells (intensity, %)		Tumor (intensity, %)		Inflammatory/stromal cells (intensity, %)	
AS-01	0	2	3	1	<1	2	3	1	<1
AS-02	0	2	<1	0	100	2	<1	0	100
AS-03	0	0	100	1	10–15	0	100	1	10–15
AS-03	0	0	100	0	100	0	100	0	100
AS-04	0	2	5	1	5	2	5	1	5
AS-05	0	0	100	0	100	0	100	0	100
AS-06	0	0	100	0	100	0	100	0	100
AS-07	0	0	0	1	15	0	0	1	15
AS-08	0	0	100	1	15	0	100	1	15
AS-09	0	2	5	1	5	2	5	1	5
AS-10	0	0	100	1	15	0	100	1	15
AS-11	0	2	5	1	15	2	5	1	10
AS-12	0	0	100	0	100	0	100	0	100
AS-13	0	2	10	0	100	2	10	0	100
AS-14	0	0	100	0	100	1	1	1	1
AS-15	0	2	1	0	100	2	1	0	100
AS-16	0	2	<1	1	5	2	3	1	5
AS-17	0	2	2	1	1	2	2	1	1
AS-18	0	2	3	0	100	2	3	0	100
AS-19	0	0	100	1	<1	0	100	1	<1
AS-20	0	2	1	0	100	2	3	0	100
AS-21	0	0	100	0	100	0	100	0	100
AS-22	0	0	100	0	100	2	<1	0	100
AS-23	0	2	1	0	100	2	1	0	100
AS-24	5.7	3	50	1	10	3	50	1	10
AS-25	0	0	100	0	100	0	100	0	100
AS-26	0	2	<1	0	100	2	1	0	100

### Association Between Tumor Cell PD-L1 Expression and VEGF-Related Genes

We quantified transcripts of VEGFA, VEGFR1, and VEGFR2 in all samples *via* Nanostring panel. The mean log2 normalized counts (minimum, maximum) were VEGFA = 8.7 (7.3, 11.7), VEGFR1 = 6.0 (0, 9.0), and VEGFR2 = 8.6 (6.2, 11.0). There was no observed significant correlation between dichotomized tumor PD-L1 expression (positive/negative) and VEGFA (*P* = 0.41), VEGFR1 (*P* = 0.65), and VEGFR2 (*P* = 0.73). There was also no significant correlation between tumor-infiltrating PD-L1/PD-1 expression and VEGF-related genes.

### Survival

A Cox model assessing overall survival in PD-L1 tumor cell or tumor-infiltrating immune cell positive primary tumors found no statistical significance [HR = 1.10 (0.22–5.51), *p* = 0.90]. Similarly, no significant differences were observed for VEGF (HR = 1.28, *p* = 0.33), VEGFR1 (HR = 0.95, *p* = 0.78), and VEGFR2 (HR = 1.09, *p* = 0.61).

### Association Between Nanostring Genes and Patient Characteristics

We applied linear mixed models to assess the association between expression of 27 genes and the patient characteristics presented in Table 1. Significant associations (*P* < 0.05) are presented (Table 3). We note a number of differences between grade, with VEGF having higher expression in high grade tumors [Fold change (FC) = 1.98, *P* = 0.03] and PLEKHA7, SFRP1, and MAOB displaying lower expression in high grade tumors (PLEKHA7 FC = 0.35, *P* = 0.02; SFRP1 FC = 0.17, *P* = 0.04; MAOB FC = 0.21, *P* = 0.04). IMP3 was higher in cutaneous tumors (FC = 1.83, *P* = 0.006) and SAA4 was lower in cutaneous tumors (FC = 0.38, *P* = 0.04). Compared to primary tumors, recurrent tumors had different expression in IMP3 (FC = 1.73, *P* = 0.02), KI-67 (FC = 3.47, *P* = 0.04), and TCN2 (FC = 0.35, *P* = 0.045); likewise, metastases had differing expression in VEGF (FC = 2.52, *P* = 0.01), IMP3 (FC = 0.60, *P* = 0.046), and TCEA3 (FC = 5.30, *P* = 0.02).

**Table 3:** Significant associations between patient characteristics and gene expression.

Gene	Covariate	Comparison	Fold change	*P*-value
IMP3	Cutaneous	Yes vs no	1.83	0.006
PLEKHA7	Grade	High vs low	0.35	0.023
Vascular endothelial growth factor (VEGF)	Grade	High vs low	1.98	0.028
SAA4	Cutaneous	Yes vs no	0.38	0.036
SFRP1	Grade	High vs low	0.17	0.036
MAOB	Grade	High vs low	0.21	0.039
VEGF	Tumor type	Metastases vs primaries	2.52	0.014
		Recurrences vs primaries	1.31	0.373
IMP3	Tumor type	Metastases vs primaries	0.60	0.046
		Recurrences vs primaries	1.73	0.016
TCEA3	Tumor type	Metastases vs primaries	5.30	0.019
		Recurrences vs primaries	0.99	0.982
KI-67	Tumor type	Metastases vs primaries	0.71	0.601
		Recurrences vs primaries	3.47	0.036
TCN2	Tumor type	Metastases vs primaries	1.51	0.482
		Recurrences vs primaries	0.35	0.045

## Discussion

The recent success in the treatment of melanoma, renal cell cancer, non-small cell lung cancer with PD-1/PD-L1 blockade has generated much interest to determine if such treatment is also applicable to other cancers. Angiosarcoma is a rare cancer without effective standard therapeutic options in the advance setting and, therefore, alternative therapies that boost the anticancer immune response, such as PD-1/PD-L1 blockade may be an effective option.

Reports for sarcoma show a PD-L1 positive expression rate that ranges from 12 to 58% (8–10). D’Angelo et al. reported tumor PD-L1 IHC expression of 12% in 50 sarcoma samples, of which, none of the 3 angiosarcoma specimens expressed tumor PD-L1 (10). Kim et al. performed a similar study and reported a positive tumor PD-L1 IHC expression rate of 58% in 105 sarcoma samples (9). In their study, 4 of 5 angiosarcoma specimens were positive for PD-L1 tumor expression. Another group reported PD-L1 expression of 43% in 82 sarcoma samples; however, none of their samples were angiosarcoma. All three studies used different PD-L1 antibodies and thresholds for positive expression.

In our study of 27 previously untreated angiosarcoma samples, we report a PD-L1-positive tumor cell and tumor-infiltrating immune cell expression rate of 19 and 33%, respectively. Multiple assays with varying definitions of PD-L1-positive report that expression of PD-L1 suggests that tumors are more responsive to anti-PD-1 therapy than those that lack expression (17–19). Our data suggest that a substantial proportion of angiosarcomas may respond to such therapy. PD-L1 is not a specific biomarker for response to PD-1 blockade; in other words, a lack of tumor PD-L1 expression does not necessarily indicate that the tumor will not respond to anti-PD-1 therapy. One reason for this is that there are technical factors, such as tissue processing and cut-off standards that determine whether a specimen is positive for PD-L1. Another reason is that tumors that may be sensitive to anti-PD-1 therapy may not necessarily have the biological pathways that lead to PD-L1 expression. Therefore, as new predictive biomarkers are developed, it is possible that a higher proportion of angiosarcomas may be eligible for anti-PD-1 therapy.

The prognostic impact of PD-L1 expression in sarcomas is not well-known. D’Angelo et al. reported no association between PD-L1 expression and overall survival (10). Two other studies did report that PD-L1 expression is an independent negative predictor of overall survival (8, 9). These studies evaluated PD-L1 expression in a variety of histologic subtypes and a total of 8 angiosarcomas were evaluated in the three studies. Shimizu et al. identified PD-L1 expression in 40% (21/52) Japanese patients with cutaneous angiosarcoma and reported that PD-L1 expression predicted worse outcomes. In contrast, this study of 27 angiosarcoma specimens from 25 Caucasian patients, PD-L1 expression in either tumor cells or tumor-infiltrating immune cells was not associated with overall survival. What accounts for this discordance is not clear. Human populations have been shown to differ in their immune responses, and, therefore, whether these differences explain the difference between Eastern and Western patient population remains to be elucidated (20, 21). Therefore, the value of PD-L1 as both a predictive and prognostic biomarker is in question, and further study will be needed to better determine the prognosis of patients based on the tumor immune microenvironment, and to identify patients who will benefit from PD-1/PD-L1 blockade therapy.

Overexpression of the VEGF pathway has been shown to be immunosuppressive (22–24). Our group previously reported an inverse relationship between PD-L1 expression and the VEGF-related genes in renal cell carcinoma. Since angiosarcomas are known to have an overexpression of VEGF-related genes, we studied the relationship between PD-L1 expression and the VEGF pathway. Interestingly, we found no relationship between the two. One explanation for this finding is that other aberrant pathways, such as TP53, can be responsible for angiosarcoma growth (3). Another explanation is that PD-L1 expression on the cell membrane can represent an oncogene-driven process or an inflammatory process (25). Oncogene-driven PD-L1 expression is typically diffuse and not associated with immune cell infiltrate. In contrast, inflammatory-driven PD-L1 expression is a component of the adaptive immune response and is associated with an immune cell infiltrate. We observed co-localization of tumor PD-L1 expression and brisk TILs in 3 out of 5 specimens. Since there appeared to be no correlation between VEGF-related gene expression and PD-L1 expression, it is possible that the PD-L1 expression observed in angiosarcomas may be secondary to an inflammatory process rather than an oncogenic one. How this impacts the value of PD-L1 expression is not entirely clear and will require further study in larger group of specimens.

A limitation of this study is technical issues associated with (1) samples date back to 1994, and, therefore, the effect of long storage time on PD-L1 expression is not known; (2) variable time to fixation could also modulate the level of PD-L1 expression; and (3) the lack of a clear definition of what defines “positive” expression and which antibody to utilize makes interpretation of these results problematic. If we were to revise our definition of “positive” to those that contained any staining, the proportion of specimens that is PD-L1 positive increases to 52% (*n* = 14). Whether such a definition would have an impact on predicting response to therapy is not known. When re-analyzed, the relationship with VEGF-related gene expression and prognosis did not change. Another set of limitations with this study center on biological issues. These include (1) the study included primary, recurrent, and metastatic angiosarcoma specimens, and although multiple studies have shown stability in expression between primary and metastatic sites (25), it is not known if PD-L1 expression evolves for angiosarcoma; (2) the intratumoral heterogeneity of PD-L1 expression is not known, and, therefore, the spatial location of the sample tested may alter our findings; and (3) finally, anti-PD-1 therapy was recently approved for any tumor with mismatch repair deficiency. It is thought that mismatch repair leads to a high tumor mutational burden, and hence greater neoantigen expression that can stimulate the immune system and thus be more affected by anti-PD-1 therapy (26). Though we did not assess mismatch repair status, a recent tumor mutation burden analysis of 100,000 human cancers revealed that angiosarcomas have a very low tumor mutational burden, suggesting that these tumors likely are mismatch repair proficient and microsatellite stable (27).

In summary, the microenvironment expresses tumor PD-L1 in a substantial proportion of angiosarcomas. The PD-L1 overexpression does not appear to be associated with the pathogenesis of angiosarcoma nor does it appear to correlate with overall survival. Yet, there still may be a role for anti-PD-1 therapy for these difficult to treat tumors. These data support the need for further study of the checkpoint pathway and potential blockade in angiosarcomas.

## Ethics Statement

This was a retrospective review of the cancer database to identify patients and specimens. Mayo Clinic Institutional Review Board approved the study prior to collecting data and performing analysis. The study was deemed as minimal risk and therefore informed consent from study participants was not obtained.

## Author Contributions

SB contributed to the conception and design of the study, experiments, analysis, and interpretation of the data, and drafting and critical revision of the article. ZG and MK contributed to collection, analysis, and interpretation of the data; critical revision of the article; and generation of the figures. TM and SA contributed to the analysis and interpretation of the data and critical revision of article. MP and DS contributed to the experiments, analysis, and interpretation of the data, and critical revision of the article. RJ contributed to the conception and design of the study, experiments, analysis, and interpretation of the data, and critical revision of the article. All authors gave final approval of the article.

## Conflict of Interest Statement

The research was conducted in the absence of any commercial or financial relationships that could be construed as a potential conflict of interest. The reviewer HD declared a shared affiliation, with no collaboration, with several of the authors, SB, DS, MP, SA, MK, RJ, to the handling editor.

## References

[1] Young RJ Brown NJ Reed MW Hughes D Woll PJ. Angiosarcoma. Lancet Oncol (2010) 11:983–91. 10.1016/S1470-2045(10)70023-1 20537949

[2] Fury MG Antonescu CR Van Zee KJ Brennan MF Maki RG. A 14-year retrospective review of angiosarcoma: clinical characteristics, prognostic factors, and treatment outcomes with surgery and chemotherapy. Cancer J (2005) 11:241–7. 10.1097/00130404-200505000-00011 16053668

[3] Zietz C Rössle M Haas C Sendelhofert A Hirschmann A Stürzl M MDM-2 oncoprotein overexpression, p53 gene mutation, and VEGF up-regulation in angiosarcomas. Am J Pathol (1998) 153:1425–33. 10.1016/S0002-9440(10)65729-X 9811333 PMC1876718

[4] Gabrilovich DI Nagaraj S. Myeloid-derived suppressor cells as regulators of the immune system. Nat Rev Immunol (2009) 9:162–74. 10.1038/nri2506 19197294 PMC2828349

[5] Gooden MJ de Bock GH Leffers N Daemen T Nijman HW. The prognostic influence of tumour-infiltrating lymphocytes in cancer: a systematic review with meta-analysis. Br J Cancer (2011) 105:93–103. 10.1038/bjc.2011.189 21629244 PMC3137407

[6] Fujii H Arakawa A Utsumi D Sumiyoshi S Yamamoto Y Kitoh A CD8(+) tumor-infiltrating lymphocytes at primary sites as a possible prognostic factor of cutaneous angiosarcoma. Int J Cancer (2014) 134:2393–402. 10.1002/ijc.28581 24243586

[7] Topalian SL Drake CG Pardoll DM. Immune checkpoint blockade: a common denominator approach to cancer therapy. Cancer Cell (2015) 27:450–61. 10.1016/j.ccell.2015.03.001 25858804 PMC4400238

[8] Kim C Kim EK Jung H Chon HJ Han JW Shin KH Prognostic implications of PD-L1 expression in patients with soft tissue sarcoma. BMC Cancer (2016) 16:434. 10.1186/s12885-016-2451-6 27393385 PMC4938996

[9] Kim JR Moon YJ Kwon KS Bae JS Wagle S Kim KM Tumor infiltrating PD1-positive lymphocytes and the expression of PD-L1 predict poor prognosis of soft tissue sarcomas. PLoS One (2013) 8:e82870. 10.1371/journal.pone.0082870 24349382 PMC3859621

[10] D’Angelo SP Shoushtari AN Agaram NP Kuk D Qin LX Carvajal RD Prevalence of tumor-infiltrating lymphocytes and PD-L1 expression in the soft tissue sarcoma microenvironment. Hum Pathol (2015) 46:357–65. 10.1016/j.humpath.2014.11.001 25540867 PMC5505649

[11] Shimizu A Kaira K Okubo Y Utsumi D Yasuda M Asao T Positive PD-L1 expression predicts worse outcome in cutaneous angiosarcoma. J Glob Oncol (2017) 3:360–9. 10.1200/JGO.2016.005843 28831444 PMC5560454

[12] Joseph RW Parasramka M Eckel-Passow JE Serie D Wu K Jiang L Inverse association between programmed death ligand 1 and genes in the VEGF pathway in primary clear cell renal cell carcinoma. Cancer Immunol Res (2013) 1:378–85. 10.1158/2326-6066.CIR-13-0042 24778130 PMC4322777

[13] Gatalica Z Snyder C Maney T Ghazalpour A Holterman DA Xiao N Programmed cell death 1 (PD-1) and its ligand (PD-L1) in common cancers and their correlation with molecular cancer type. Cancer Epidemiol Biomarkers Prev (2014) 23:2965–70. 10.1158/1055-9965.EPI-14-0654 25392179

[14] Joneja U Vranic S Swensen J Feldman R Chen W Kimbrough J Comprehensive profiling of metaplastic breast carcinomas reveals frequent overexpression of programmed death-ligand 1. J Clin Pathol (2017) 70:255–9. 10.1136/jclinpath-2016-203874 27531819 PMC5339564

[15] Frishberg DP Balch C Balzer BL Crowson AN Didolkar M McNiff JM Protocol for the examination of specimens from patients with melanoma of the skin. Arch Pathol Lab Med (2009) 133:1560–7. 10.1043/1543-2165-133.10.1560 19792045

[16] Brumbaugh CD Kim HJ Giovacchini M Pourmand N. NanoStriDE: normalization and differential expression analysis of NanoString nCounter data. BMC Bioinformatics (2011) 12:479. 10.1186/1471-2105-12-479 22177214 PMC3273488

[17] Topalian SL Hodi FS Brahmer JR Gettinger SN Smith DC McDermott DF Safety, activity, and immune correlates of anti-PD-1 antibody in cancer. N Engl J Med (2012) 366:2443–54. 10.1056/NEJMoa1200690 22658127 PMC3544539

[18] Taube JM Klein A Brahmer JR Xu H Pan X Kim JH Association of PD-1, PD-1 ligands, and other features of the tumor immune microenvironment with response to anti-PD-1 therapy. Clin Cancer Res (2014) 20:5064–74. 10.1158/1078-0432.CCR-13-3271 24714771 PMC4185001

[19] Powles T Eder JP Fine GD Braiteh FS Loriot Y Cruz C MPDL3280A (anti-PD-L1) treatment leads to clinical activity in metastatic bladder cancer. Nature (2014) 515:558. 10.1038/nature13904 25428503

[20] Nédélec Y Sanz J Baharian G Szpiech ZA Pacis A Dumaine A Genetic ancestry and natural selection drive population differences in immune responses to pathogens. Cell (2016) 167:657–669.e21. 10.1016/j.cell.2016.09.025 27768889

[21] Quach H Rotival M Pothlichet J Loh YE Dannemann M Zidane N Genetic adaptation and neandertal admixture shaped the immune system of human populations. Cell (2016) 167:643–656.e17. 10.1016/j.cell.2016.09.024 27768888 PMC5075285

[22] Huang Y Goel S Duda DG Fukumura D Jain RK. Vascular normalization as an emerging strategy to enhance cancer immunotherapy. Cancer Res (2013) 73:2943–8. 10.1158/0008-5472.CAN-12-4354 23440426 PMC3655127

[23] Wei J Wu A Kong LY Wang Y Fuller G Fokt I Hypoxia potentiates glioma-mediated immunosuppression. PLoS One (2011) 6:e16195. 10.1371/journal.pone.0016195 21283755 PMC3024401

[24] Facciabene A Peng X Hagemann IS Balint K Barchetti A Wang LP Tumour hypoxia promotes tolerance and angiogenesis via CCL28 and T(reg) cells. Nature (2011) 475:226–30. 10.1038/nature10169 21753853

[25] Patel SP Kurzrock R. PD-L1 expression as a predictive biomarker in cancer immunotherapy. Mol Cancer Ther (2015) 14:847–56. 10.1158/1535-7163.MCT-14-0983 25695955

[26] Le DT Uram JN Wang H Bartlett BR Kemberling H Eyring AD PD-1 blockade in tumors with mismatch-repair deficiency. N Engl J Med (2015) 372:2509–20. 10.1056/NEJMoa1500596 26028255 PMC4481136

[27] Chalmers ZR Connelly CF Fabrizio D Gay L Ali SM Ennis R Analysis of 100,000 human cancer genomes reveals the landscape of tumor mutational burden. Genome Med (2017) 9:34. 10.1186/s13073-017-0424-2 28420421 PMC5395719

